# Temperature and concentration calibration of aqueous polyvinylpyrrolidone (PVP) solutions for isotropic diffusion MRI phantoms

**DOI:** 10.1371/journal.pone.0179276

**Published:** 2017-06-19

**Authors:** Friedrich Wagner, Frederik B. Laun, Tristan A. Kuder, Anna Mlynarska, Florian Maier, Jonas Faust, Kerstin Demberg, Linus Lindemann, Boris Rivkin, Armin M. Nagel, Mark E. Ladd, Klaus Maier-Hein, Sebastian Bickelhaupt, Michael Bach

**Affiliations:** 1 Medical Physics in Radiology, German Cancer Research Center (DKFZ), Heidelberg, Germany; 2 Institute of Radiology, University Hospital Erlangen, Erlangen, Germany; 3 Radiology, German Cancer Research Center (DKFZ), Heidelberg, Germany; 4 Medical and Biological Informatics, German Cancer Research Center (DKFZ), Heidelberg, Germany; NIH, UNITED STATES

## Abstract

To use the “apparent diffusion coefficient” (*D*_app_) as a quantitative imaging parameter, well-suited test fluids are essential. In this study, the previously proposed aqueous solutions of polyvinylpyrrolidone (PVP) were examined and temperature calibrations were obtained. For example, at a temperature of 20°C, *D*_app_ ranged from 1.594 (95% CI: 1.593, 1.595) μm^2^/ms to 0.3326 (95% CI: 0. 3304, 0.3348) μm^2^/ms for PVP-concentrations ranging from 10% (w/w) to 50% (w/w) using K30 polymer lengths. The temperature dependence of *D*_app_ was found to be so strong that a negligence seems not advisable. The temperature dependence is descriptively modelled by an exponential function exp(*c*_2_ (*T* − 20°*C*)) and the determined *c*_2_ values are reported, which can be used for temperature calibration. For example, we find the value 0.02952 K^-1^ for 30% (w/w) PVP-concentration and K30 polymer length. In general, aqueous PVP solutions were found to be suitable to produce easily applicable and reliable *D*_app_-phantoms.

## Introduction

Diffusion weighted imaging (DWI) is a magnetic resonance imaging (MRI) technique that finds widespread application today [1–4]. By means of diffusion-weighting magnetic field gradient pulses [5], the diffusion of water in human tissue can be measured in a spatially resolved manner thus yielding, for example, maps of the apparent diffusion coefficient [1]. Cellular structures restrict the diffusive motion, which allows inferring information about structural properties of cells and tissue. This can be exploited, for example, to depict the course of white matter tracts in the human brain by measuring anisotropic diffusion with diffusion tensor imaging (DTI) [6] and with fiber tracking [7, 8]. More advanced methods like q-ball imaging [9] and diffusion kurtosis imaging [10] extend the diffusion tensor approach making possible the detection of fiber crossings and the detection of non-Gaussian diffusion properties.

As DWI is a sensitive technique, several approaches for validation using ground truth phantoms have been proposed. On the one hand, phantoms mimicking anisotropic diffusion of neuronal tissue have been proposed. These phantoms are often made using thin fibers that make the diffusive motion of the fiber-embedding fluid anisotropic. Such phantoms have been used for multiple different methodological developments in the fields of diffusion tensor imaging [11–21], kurtosis tensor imaging [12, 22], q-ball imaging [20, 21, 23–26], tract-based spatial statistics [27], and fiber tracking [13, 20, 28]. Besides the development of advanced DWI methods, also the quality and repeatability [16] was assessed with such phantoms as well as the comparability of tensor metrics in a multi-center study [29]. Moreover, phantoms consisting of hollow fibers and capillaries have been proposed and were applied successfully (e.g. [30–34]).

Stepping back from these advanced applications, an increased interest has recently sparked in finding good isotropic diffusion phantoms. While water has been used in a majority of cases [35–40], it has some drawback such as a low viscosity, which may result in non-negligible fluid convection, or a diffusion coefficient that is much higher than that of typical human tissues and lesions. Consequently, a number of further test fluids has been proposed such as cyclohexane [41, 42], dioxane [41], DMSO [41], pentanol [41], sucrose [43], ethylene Glycol [44], cyclohexane [45], cycloheptane [42, 45], cyclooctane [42, 45], n-octane to n-hexadecane [41, 42], ethanol [42], n-propanol [42], n-butanol [42], and aqueous solutions of polyvinylpyrrolidone (PVP) [46–50]. Applications included quality control examinations and investigations of precision and accuracy [35, 45, 51, 52], evaluations of gradient performance [44], sequence evaluation [46].

One issue with all test fluids is that their diffusion properties depend on their temperature. One possibility is to fix the temperature, for example, by putting the test fluid in ice water thus maintaining a constant temperature of 0°C [38, 39, 51]. The other approach is to correct the measured diffusion coefficients to a reference temperature using calibration curves [35, 36, 41, 42, 44, 45]. As PVP solutions have many favorable properties, which comprise a concentration-adjustable water diffusion coefficient and an increased viscosity of the fluid, the aim of this work was to provide the calibration curves for two common PVP polymer chain lengths for typical bore temperature of clinical whole body MR scanners.

## Material and methods

### Phantom

**Fig 1:**
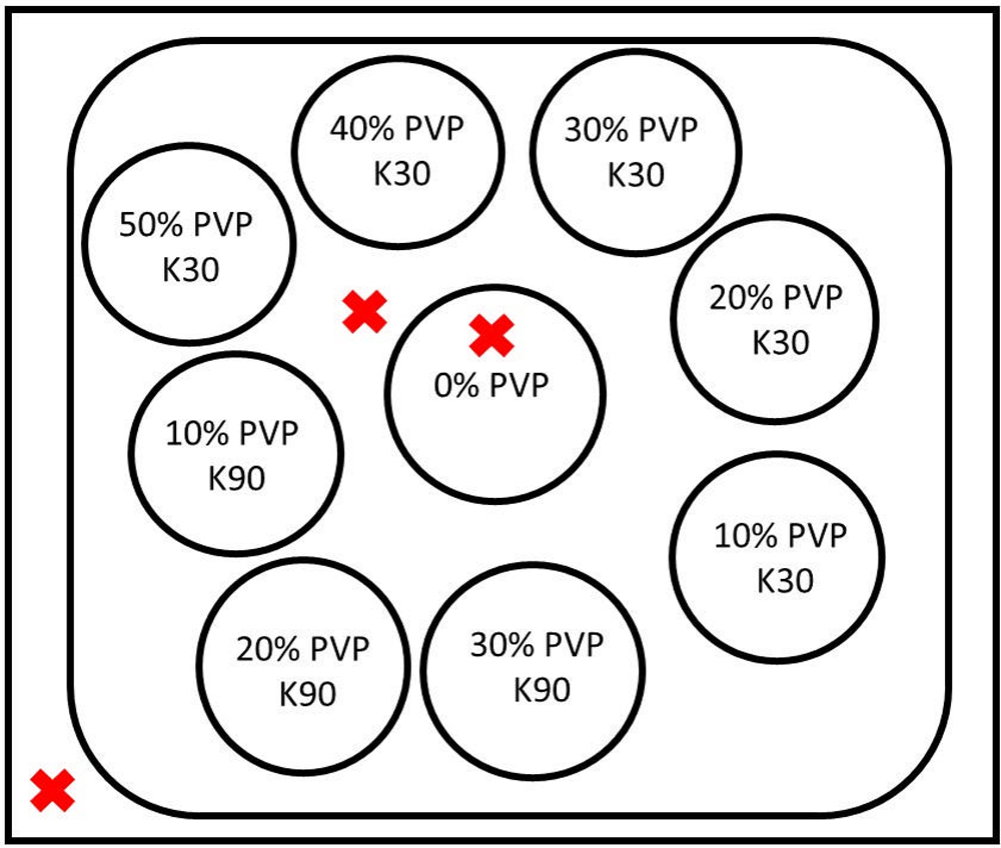
(single column). Sketch of the phantom. Tubes with different PVP solutions were contained in a water-filled bin, which was itself immersed in a water bath. Temperature was recorded at three positions (red crosses).

Nine plastic tubes (volume 50 ml) were filled with aqueous solutions of polyvinylpyrrolidone (PVP) (AppliChem GmbH, Darmstadt, Germany, K30 with 44–55 kg/mol and K90 with 1,000–1,500 kg/mol) with concentrations *c*_PVP_ of 10, 20, 30, 40, and 50% for K30 and 10, 20, and 30% for K90 (w/w). Concentrations of K90 of 40% and 50% were not used, since these concentrations resulted in fluids of high viscosity with trapped air bubbles. The tubes were immersed in a water-filled closed bin, which was placed in a surrounding water bath (Fig 1) to fill the used receive coil to a maximal extent. The temperature was recorded using a fiber optical thermometer (Luxtron FOT Lab Kit, LumaSense Technologies, CA, USA) in intervals of approximately 1 s at three positions: In the central water-filled tube, in the bin and in the surrounding water bath (see red crosses in Fig 1). The temperature of the central water-filled probe was used; the other two probes were used for an additional verification. Moreover, the recorded temperature was averaged over the scan time of each measurement sequence. The vendor provided accuracy is ±0.5°C within 50°C of the calibration temperature, ±0.2°C within 20°C of calibration temperature, and ±0.1°C at calibration temperature. The phantom was calibrated with a one-point temperature calibration using an ice-water bath before each measurement series. Verification exams of the Luxtron thermometer revealed a temperature offset of approximately +0.9°C (see supplemental material S1 File). The recorded temperature values were thus corrected by subtracting this offset.

### MRI experiments

MRI experiments were performed on a 1.5 T scanner (Magnetom Aera, Siemens Healthcare, Erlangen, Germany) using a 20-channel head-coil and on a 3.0 T scanner (Magnetom Prisma fit, Siemens Healthcare, Erlangen, Germany) using a 64-channel head coil. A diffusion-weighted echo planar imaging sequence was used and data was acquired with two parameters sets:

1. High resolution parameter set: repetition time TR = 5000 ms, echo time TE = 144 ms, readout bandwidth = 1000 Hz/px, field of view = 200 x 200 mm^2^, 20 slices, voxel size = 1 x 1 x 5 mm^3^, parallel imaging with GRAPPA and acceleration factor of 2, three orthogonal, monopolar diffusion weightings, strength of diffusion weighting *b* = 0, 100, 200, 300, 400, 500, 600, and 700 s/mm^2^ acquired with 1 average (*b* = 0 s/mm^2^), 2 averages (b = 100 to 500 s/mm^2^), or three averages (b = 600 and 700 s/mm^2^), magnitude images were averaged, the vendor-provided image filter was set to “strong” to suppress Gibbs ringing. Acquisition time was 5:52 min.
2. Low resolution parameter set: Identical parameters as for the high resolution parameter set except for: TE = 86 ms, voxel size = 2 x 2 x 5 mm^3^.

The tubes were oriented perpendicular to the main magnetic field, i.e. along the anterior-posterior direction, and the image plane was coronal, i.e. perpendicular to the tubes. High and low resolution datasets were acquired alternatingly. A low duty cycle sequence of 8:22 min was inserted between them to avoid potential heating of the gradient system.

Four data acquisition series were run at each scanner. Two times, the phantom was warmed up to about 30° and was let to cool down. Two times, the phantom was cooled to about 14°C and was let to warm up. Each of the series was acquired in an overnight scan lasting about 10 to 12 hours. One temperature recording file of a 3.0 T run was corrupted so that this run was excluded from the analysis.

### Data evaluation

**Fig 2:**
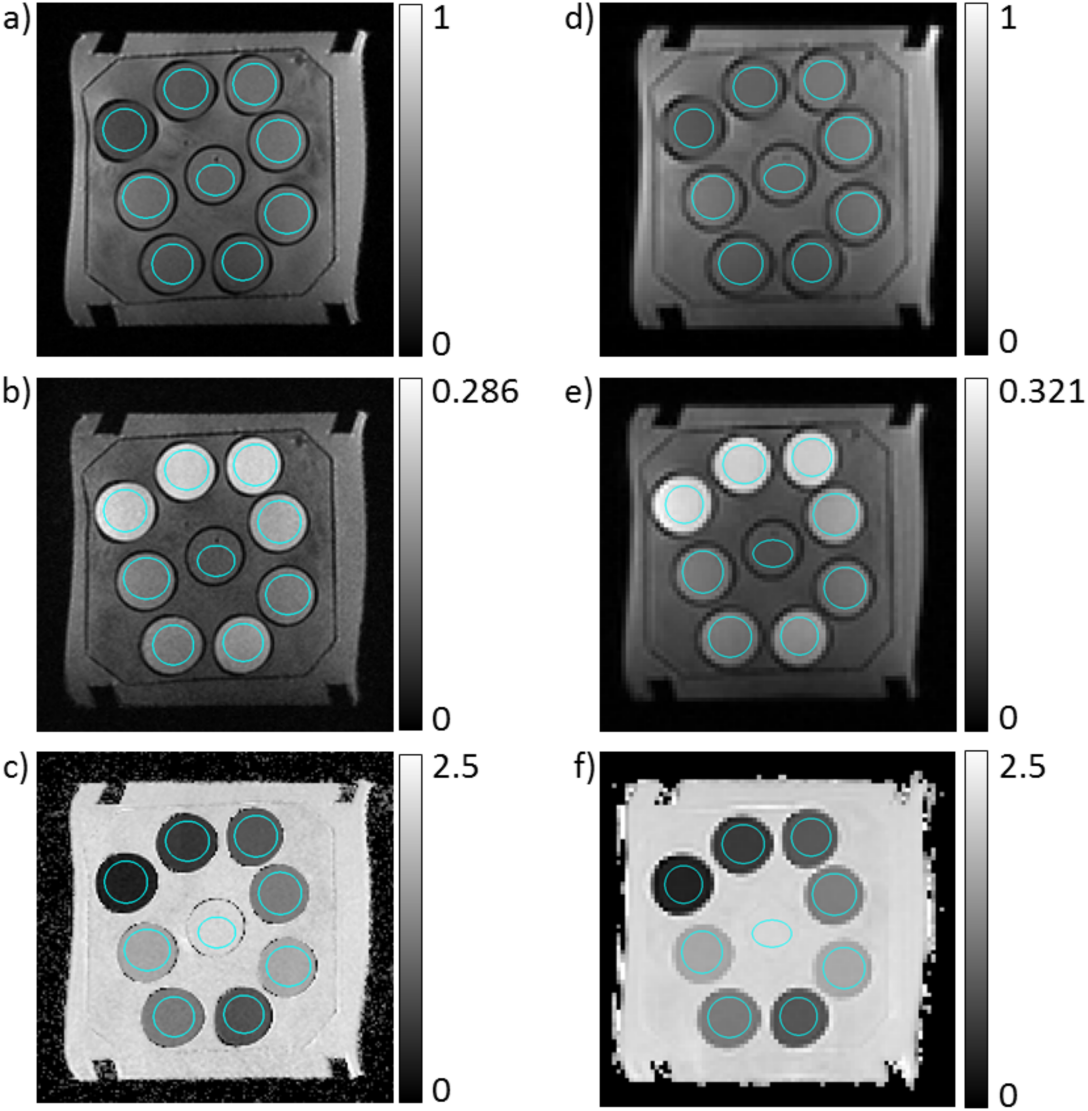
(double column): Images of the used phantom. a,b,c) High resolution images. d,e,f) Low resolution images. a,d) Signal images without diffusion weighting, i.e. with *b* = 0 s/mm^2^. b,e) Signal image with *b* = 700 s/mm^2^. Signal images are min-max normalized. c,f) *D*_app_-maps of the phantom in units of μm^2^/ms. Cross sections of the volumes of interest are marked in blue in a and d.

Apparent diffusion coefficients (*D*_app_) were computed for each voxel using the equation

$$S\left(b\right)=S_{0}\text{exp}\left(-bD_{\text{app}}\right),$$ (1)

where *S*(*b*) is the signal intensity in dependence of the b-value with the free fit parameters *S*_0_ and *D*_app_ [5, 6]. Mean *D*_app_-values were computed for manually drawn volumes of interest (VOIs). The circular cross section was specified on the images within the tubes with a safety margin of roughly 3 mm to the tube boundary (see Fig 2). In slice direction, the VOIs encompassed 5 or 6 adjacent slices. VOIs were drawn such that regions of increased distortions at the edges of the tubes were spared. The diffusion weighted maps were inspected visually to avoid regions with other potential artifacts.

The dependency of *D*_app_ on the temperature *T* was descriptively modelled by the equation

$$D_{\text{app}}=c_{1}\cdot \text{exp}\left(c_{2}\cdot \left(T-T_{0}\right)\right).$$ (2)

Here, *c*_1_ is the apparent diffusion coefficients at *T*_0_ = 20°C and *c*_2_ describes the temperature dependence. Eqs 1 and 2 were fitted using the Levenberg-Marquardt algorithm thus determining *D*_app_ and *S*_0_ (not used in further calculation) in Eq 1, and *c*_1_ and *c*_2_ in Eq 2. 95% confidence intervals were computed.

The dependence of *c*_2_ on *c*_PVP_ was modelled by means of a linear fit according to the equation

$$c_{2}=a_{c_{2}}+b_{c_{2}}\cdot c_{\text{PVP}}$$ (3)

The coefficients $a_{c_{2}}$ and $b_{c_{2}}$ were determined by means of a linear least square fit using the values determined for *c*_2_ with fitting weights equal to the inverse of the 95% confidence intervals. Additionally, aiming at a further increase of accuracy, the value for *c*_2_ at *c*_PVP_ = 0 from Mill et al. [53] was included in the fit with a fitting weight equal to the average weight of the other data points.

An estimate of the signal to noise ratio (SNR) was obtained by dividing the signal in the PVP solution by the signal in a region of interest outside the phantom. One has to note that this estimate has to be considered being far from accurate, in part due to the use of parallel imaging.

## Results

An image slice of the phantom is shown in Fig 2 for one high and one low resolution image set acquired at 1.5 T. Images acquired with *b* = 0 s/mm^2^ (Fig 2a and 2d), with *b* = 700 s/mm^2^ (Fig 2b and 2e), and maps of *D*_app_ (Fig 2c and 2f) are shown. A negative correlation between PVP concentration and *D*_app_ is observed in the *D*_app_-maps. In the *b* = 700 s/mm^2^ images, higher PVP concentrations are reflected by a reduced signal drop. The estimated SNR ranged from 40 to 140 in the *b* = 0 images.

**Fig 3:**
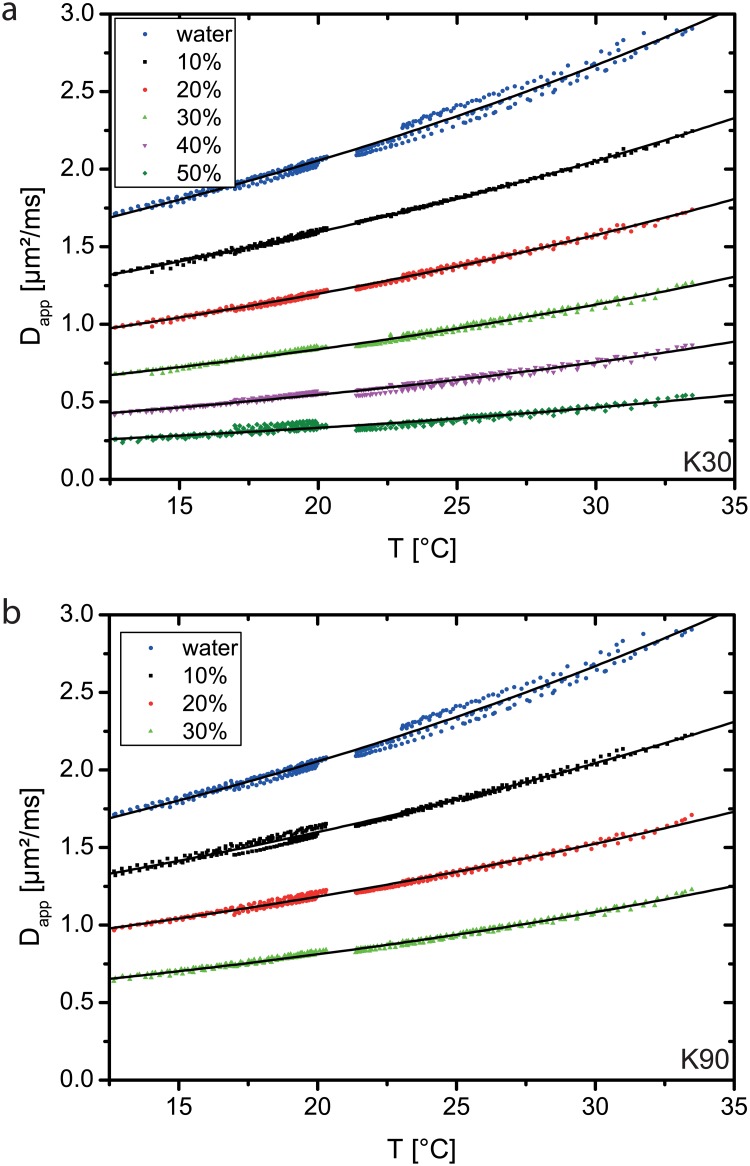
(single column): Dependence of apparent diffusion coefficient *D*_app_ on temperature for K30 and K90. Markers represent measured values and lines represent fits according to Eq 2.

Fig 3 shows the dependency of *D*_app_ on the temperature *T* for K30 and for K90. Fits of Eq (2) are shown as black lines and are generally in good accordance with the measured data. The data of the seven displayed acquisition runs are generally also in good agreement, but few outlier runs are present, e.g. for water and for K30 with *c*_PVP_ = 50%. Tables 1 and 2 provide the fitted parameters *c*_1_ and *c*_2_. The differences of the fit-parameters between the two K-values at one specific temperature are rather low. Parameters *c*_1_ and *c*_2_ that were determined only with the 1.5T scanner, or with the 3.0T scanner, are provided as supplemental material S2 File.

**Table 1:** Fit parameters describing the dependency of *D*_app_ on the temperature (see Eq (2)) for K30. 95% confidence intervals are stated in brackets.

*c*_PVP_ [% (w/w)]	*c*_1_ [μm^2^/ms]	*c*_2_ [1/K]
0	2.055 (2.050, 2.059)	0.02617 (0.02583, 0.02652)
10	1.594 (1.593, 1.595)	0.02531 (0.02519, 0.02542)
20	1.197 (1.195, 1.198)	0.02749 (0.02729, 0.02769)
30	0.8388 (0.8372, 0.8403)	0.02952 (0.02921, 0.02983)
40	0.5465 (0.5447, 0.5482)	0.03247 (0.03194, 0.03299)
50	0.3326 (0.3304, 0.3348)	0.03303 (0.03197, 0.03409)
0 (Data by Mills, determined using T = 15, 25, and 35°C)	2.02 (1.864, 2.176)	0.02466 (0.01777, 0.03155)

**Table 2:** Fit parameters describing the dependency of *D*_app_ on the temperature (see Eq (2)) for K90. 95% confidence intervals are stated in brackets.

*c*_PVP_ [% (w/w)]	*c*_1_ [μm^2^/ms]	*c*_2_ [1/K]
10	1.599 (1.596, 1.601)	0.02453 (0.02428, 0.02479)
20	1.183 (1.181, 1.185)	0.02536 (0.0251, 0.02563)
30	0.8112 (0.8096, 0.8128)	0.02893 (0.02861, 0.02925)

**Fig 4:**
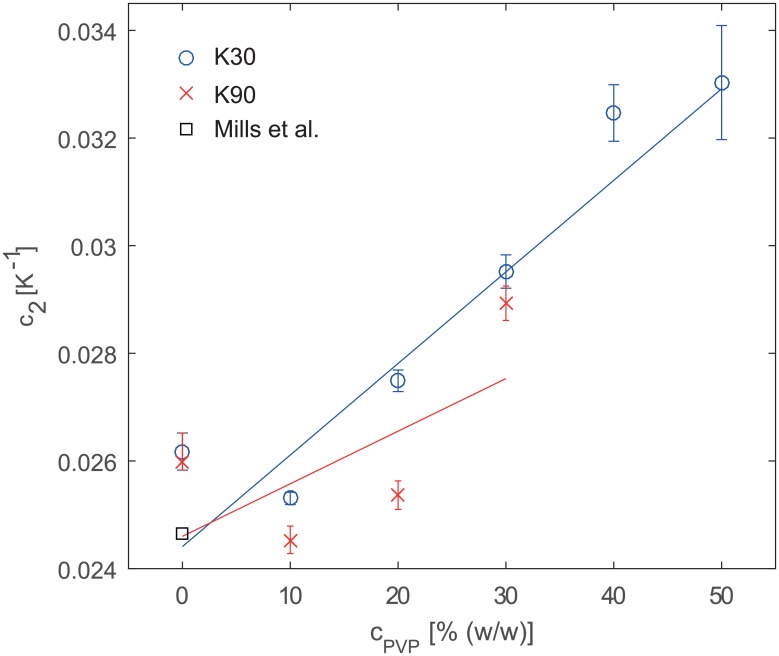
(single column): Temperature calibration coefficient *c*_2_ in dependence of PVP concentration for K30 and K90. Error bars denote 95% confidence intervals. The straight lines represent linear fits with weights inversely proportional to the width of the 95% confidence interval.

Fig 4 shows *c*_2_ in dependence of *c*_PVP_. The straight lines represent Eq (3), i.e. a linear interpolation. The coefficients are $a_{c_{2}}$ = 0.0244 K^-1^ and $b_{c_{2}}$ = 1.7011·10^−4^ K^-1^/%(w/w) for K30 and $a_{c_{2}}$ = 0.0246 K^-1^ and $b_{c_{2}}$ = 9.772·10^−5^ K^-1^/%(w/w) for K90. The data points do not lie perfectly on the interpolating straight line. If the assumption that the relation between *c*_2_ and *c*_PVP_ is linear is correct, then the linear interpolation presumably averages out experimental errors and hence it would be more advisable to use the *c*_2_ values predicted by the interpolation. These values are stated in Table 3.

**Table 3:** Recommended temperature calibration coefficient *c*_2_ in K^-1^.

*c*_PVP_ [% (w/w)]	K30	K90
0	0.0244	0.0246
10	0.0261	0.0256
20	0.0278	0.0266
30	0.0295	0.0275
40	0.0312	
50	0.0329	

## Discussion

The here provided data can be used to calibrate measurements to the sample temperature. Measurements without temperature calibration or temperature fixation seem not advisable, because the observed temperature dependency appears too strong to be acceptable.

For the case of water, the obtained coefficients *c*_1_ and *c*_2_ are in good keeping with published values. For example, using our measured *c*_1_ and *c*_2_ in Table 1 for free water, one obtains diffusion coefficients estimated at temperatures 15°C, 25°C, and 35° of 1.819 μm^2^/ms, 2.322 μm^2^/ms, and 2.963 μm^2^/ms. This matches, for example, closely to the values published by Mills, which are 1.777 μm^2^/ms, 2.299 μm^2^/ms and 2.919 μm^2^/ms [53]. Pullens et al. and Pierpaoli et al. published values for K30 at 21°C and at 22°C, but used the unit weight per volume (w/v). At 30% w/v, Pullens et al. obtained a *D*_app_ of approximately 0.852 μm^2^/ms at 21°C, and Pierpaoli et al. obtained approximately 0.955 μm^2^/ms at 22°C. Our values for *c*_1_ and *c*_2_ predict 0.960 μm^2^/ms at 21°C and 0.983 μm^2^/ms at 22°C taking into account the difference between (w/w) and (w/v) by means of linear interpolation and using calibration curves that we obtained (K30: *c*_PVP(w/v)_ = 1.0955*c*_PVP(w/w)_. K90: *c*_PVP(w/v)_ = 1.0535*c*_PVP(w/w)_): We found that 30% (w/v) corresponds to 27.4% (w/w) for K30 (see supplemental material S2 File) and used this value for calculation of *D*_app_. Thus our values are closer to those reported by Pierpaoli et al. with a difference of about 3%. Keenan et al. have reported diffusion coefficients for different PVP concentrations [48]. They used a slightly different polymer weight, but given the little difference of the coefficients *c*1 and *c*2 between K30 and K90 despite a vastly different typical molecular weight, it seems reasonable to compare their values to our K30 values. Keenan et al. report for 10% w/w that *D*_app_ ranges from 1.536 μm^2^/ms at 17.91°C to 1.714 μm^2^/ms at 22.11°C, while we find corresponding values of 1.509 μm^2^/ms to 1.684 μm^2^/ms. At 40% (w/w), Keenan et al. report that *D*_app_ ranges from 0.557 μm^2^/ms at 17.91°C to 0.640 μm^2^/ms at 22.11°C, while we find corresponding values of 0.512 μm^2^/ms to 0.584 μm^2^/ms. Thus at lower concentrations, a good agreement is observed, but at larger concentrations, the deviation becomes as large as 10%. One explanation might be that PVP is hygroscopic (maximal water content of 5% according to vendor). Thus the weight of the powder can depend on its water content. Potentially, this must be accounted for when using PVP solutions as a DWI reference standard. Interestingly, however, the ratio between *D*_app_ values at the largest and the smallest temperature is 0.640/0.557≈1.149 for Keenan et al.’s values and 0.584/0.512≈1.141 for our values indicating that the *c*_2_-based calibration by Eq (2) would be in good keeping with what one would expect using Keenan et al.’s values.

In comparison to other proposed test fluids, aqueous solutions of PVP have several advantages: First, multiple image ghosts arising from chemical shifts are not observed in the echo planar data, which would be the case, e.g., using other proposed test fluids as ethanol or n-propanol [42]. Second, the water diffusivity can be adjusted specifically to desired values by changing the PVP concentration, which is not possible, e.g. using cyclo-alkanes [41, 42, 45]. Third, one measures water protons and not protons bound to other molecules as for most of the other test fluids. As the Larmor frequency of the water protons in the PVP solution is close to the water frequency in pure water, fat suppression techniques can be tested conveniently when adding a fat containing volume to the phantom. This would be difficult, e.g., using ethanol. Fourth, unlike ethanol and alkanes, aqueous PVP solutions are not flammable. Fifth, PVP has a mono-exponential signal decay with respect to *b* and, sixth, it is independent of diffusion time [49]. Seventh, PVP solutions have a higher viscosity than pure water, which prevents fluid convection and hence increases measurement accuracy and precision. Very high PVP concentrations are, however, hard to work with, which might explain the outliers observed for *c*_PVP_ = 50%. Moreover, high PVP concentrations are more likely to contain air pockets. In particular, the K90 solutions with *c*_PVP_ = 30% appear to us to be potentially outside the trustable range due their very high viscosity and thus difficult handling. One drawback of PVP solutions is their susceptibility to mold and bacteria growth, which they have in common with many gels [54]. This can, however, be overcome by adding preservatives like sodium azide [47, 49]. One further property of increased PVP concentrations is the reduced *T*_1_ time [48, 55].

A recently proposed alternative test fluid is decamethylcyclopentasiloxane [56]. It shares some of the advantageous properties of PVP, such as non-toxicity, a single chemical shift line and mono-exponential signal decay. Its diffusion coefficient is smaller by roughly a factor of 10 compared to water, which can be an advantage in applications involving strong diffusion weightings. A drawback is that the diffusion coefficient cannot be adjusted as for aqueous PVP solutions by changing PVP concentration. Moreover it has a different chemical shift than water protons making it potentially difficult to assess fat saturation techniques.

A drawback of the current study is that the calibration curves were obtained with systems that have, of course, their own systematic errors. This was partly overcome by using two systems and several acquisition series. However, in order to perform a temperature calibration, one does not necessarily have to rely on the absolute *D*_app_ values being correct. Only the relative change with temperature, i.e. *c*_2_, must be known.

A further limitation is that the test tubes did only cover a limited space. Thus one must rely on the assumption that the measured *D*_app_ value shows little spatial variation and that ringing artifacts can be neglected (see supplemental material S4 File for an assessment of Gibbs ringing). Moreover, the rather complicated structure of the measurement setup is not ideal to acquire echo planar images. A more suitable setup addressing these two issues would have been the consecutive measurement of the different PVP solutions in spherical bins; but owing to the arising large demands on acquisition time, the more practical approach of combing all PVP solutions in a single phantom was used in this work. Another limitation and source of uncertainty is the lack of a rigorous temperature control, e.g. by a heat controlled water bath, which makes necessary the assumption that the temperature in the central bin equals the temperature of all bins.

Besides the applications mentioned in the introduction, the use of test fluids in a study may allow the investigator to come to a more quantitative experiment, which is beneficial in many regards, e.g. it is beneficial for the application of radiomics or machine learning techniques [57–59], in multi-center studies, for quantitative measures in standardized examinations, and for the definition of cut-off values.

In conclusion, reference curves were presented that can be used to calibrate *D*_app_ measurements using aqueous PVP solutions at different sample temperatures. This shall enable a more widespread use of PVP solutions as test fluids in phantoms in diffusion weighted imaging experiments.

## Supporting information

S1 File Evaluation of the accuracy of temperature measurements with Luxtron FOT Lab Kit. (PDF)

S2 File Separate evaluation for 1.5 T and 3 T. (DOCX)

S3 File PVP: Weight per weight & weight per volume. (XLSX)

S4 File Assessment of image blurring and Gibbs ringing. (DOCX)

## Abbreviations

*b*: b-value
*c*_PVP_: concentration of PVP in % (weight/weight)
*D*_app_: apparent diffusion coefficient
DWI: diffusion weighted imaging
MRI: magnetic resonance imaging
PVP: polyvinylpyrrolidone
*T*: temperature
