# Supplementary material for: Temperature and concentration calibration of aqueous polyvinylpyrrolidone (PVP) solutions for isotropic diffusion MRI phantoms
Source: PLoS One. 2017 Jun 19;12(6):e0179276. doi: 10.1371/journal.pone.0179276 (PMC5476261; doi:10.1371/journal.pone.0179276)
Supplement: S1 File — (PDF) [file pone.0179276.s001.pdf]

Supplement 1: Evaluation of the Accuracy of Temperature Measurements with Luxtron FOT Lab Kit

April 4, 2017

The accuracy of the Luxtron FOT Lab Kit fiber optic thermometer device was evaluated for temperatures in the range from 15 °C to 35 °C. Additionally, the immunity of the device to the presence of magnetic fields was verified.

1 Introduction

Accurately measured temperatures are crucial for the evaluation of the data shown in the manuscript. However, there are several challenges to perform accurate temperature measurements during an MR scan. The strong magnetic fields inside the bore of the scanner do not allow the usage of conventional electronic thermometers. One way to overcome those obstacles is to use optical thermometers, e.g. the Luxtron FOT Lab Kit. In this supplement, the accuracy of this device is examined by comparing it to two different reference thermometers. We also investigated a possible field dependency of the measured temperature values.

2 Materials

2.1 Haake F6 circulator

To compare different thermometers, an isotropic and suitable measurement environment is required. For this purpose, we used the Haake F6 circulator. This device allowed to dynamically regulate the temperature of a water bath. The setup of the experiment is shown in Figure 1.

2.2 Greisinger GTH 175/Pt

The GTH 175/PT is a resistance thermometer device which comes with a penetration probe. It offers a resolution of 0.1 °C and has an accuracy of ±0.1% of the measured value ±2 digit.

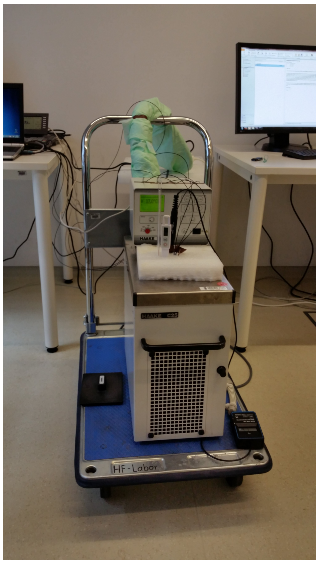

Figure 1: Setup of the experiment including the HAAKE F6 CIRCULATOR and four different thermometers.

2.3 Voltcraft DET3R

The VOLTCRAFT DET3R is also a resistance thermometer with an expected accuracy of ±0.5 °C. The VOLTCRAFT DET3R offers an optional one-point calibration which was not used in this experiment.

2.4 Luxtron FOT Lab Kit

The LUXTRON FOT LAB KIT is a fiber optic thermometry system which can measure temperatures by exciting a phosphorescent sensor which is attached at the end of a fiber optic probe. Up to four fibers can be used, all measuring the temperature separately. It offers an one-point calibration, which was done with ice water directly before setting up the experiment. The manufacturer estimates an accuracy of ±0.5 °C within 50 °C of the calibration temperature.

3 Methods

3.1 Accuracy of the Luxtron device

The setup consisted of the regulated water bath and the thermometers measuring the temperature of the water. The control unit of the HAAKE device and the LUXTRON device were connected to a computer. A program recorded the measured temperatures of all four fibers every second. The initial temperature of the water was 15 °C. In intervals of three minutes, the temperature was increased by 1 °C by the control device. The set-point temperature is shown in Figure 2. At the end of every interval, which was

acoustically indicated by the software, the temperature was also measured with the two resistance thermometers manually.

Thermal noise was visible on the measured values of the four fibers (up to a peak-to-peak amplitude of $\approx 0.7\,°C$). Therefore, the mean value of the last 30 values of each 3 minute interval was taken. Standard deviation was also calculated. For the errors on the other thermometers, please refer to section 2.

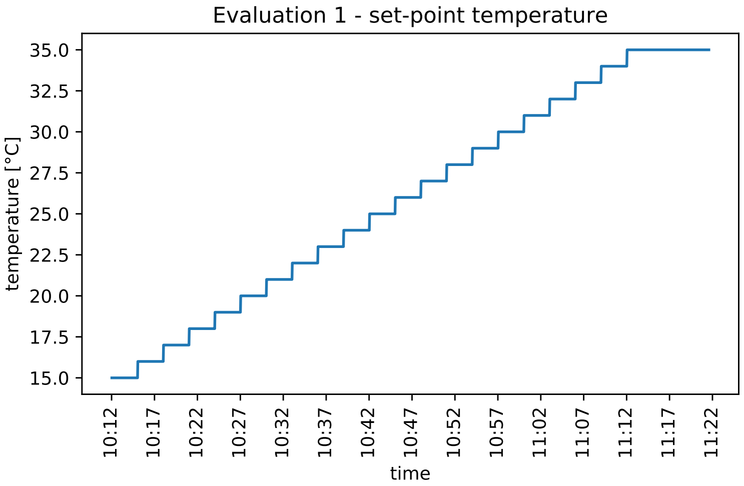

Figure 2: The set-point temperature was changed every three minutes.

3.2 Potential field dependency of the Luxtron device

The aim of the second part of the experiment was to investigate a potential impact of a magnetic field on the temperature measurements with the Luxtron device. We used the magnetic field generated by a 1.5 T scanner. To have a temperature controlled environment inside the bore of the scanner, we could not use the Haake water bath because of its ferromagnetic components. Ice water in an insulated container was used instead. During the experiment, the insulated container was slowly moved into the bore of the scanner. The temperature was recorded with the computer every second.

4 Results

4.1 Accuracy of the Luxtron device

The recorded temperatures are displayed in table 1. Because the Luxtron device was calibrated at 0 °C, we expected all fibers to reproduce that value when again exposed to ice water. The GTH 175/PT device always displayed a value which was 0.1 °C above the set-point value while the Voltcraft DET3R almost always showed a value which was 0.2 °C below the set-point temperature. Figure 3 shows, that for all measured values,

there was a positive offset of the values collected by the LUXTRON device to the values collected by the resistance thermometers. The resistance thermometer measurements indicate that the temperature of the water bath was in good agreement with the set-point temperature. Therefore, the offsets between the measured temperatures by the LUXTRON device to the actual set-point values are shown in table 2.

If you assume that the set-point values were ground truth, plotting the measured values against the set-point values and fitting a straight line to the data will result in a line through the origin. If a one-point calibration failed and a fit still produced a line through the origin but with an alternative slope, the recorded values could be corrected by performing a two-point calibration. To check this hypothesis, a linear function

$$y = a * x + b \tag{1}$$

was fitted to the data of each fiber. Figure 4 shows this exemplary for fiber 1. Table 3 shows the fitting parameters for the other fibers. The slope was about 1. However, the y-intercept was not zero. Therefore, it was not a line through the origin and a two-point-calibration would not be a solution.

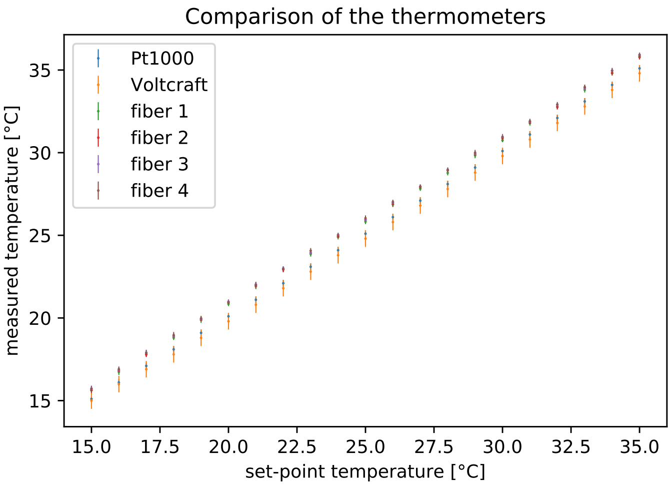

Figure 3: The LUXTRON thermometer shows an offset to the resistance thermometers.

4.2 Potential field dependency of the Luxtron device

Figure 5 shows exemplarily the plot of the temperatures measured with fiber 1 against the time. During the experiment, the insulated container filled with ice water was pushed into the MR scanner. The fibers were in the ice water during this experiment. No effect of the magnetic field on the measured temperature was visible. For verification, mean and standard deviation of the first and last 30 values of the experiment were calculated (table 4). All values lie within one sigma of the zero degrees point. We concluded that the magnetic field has no or only a very small effect on the LUXTRON device.

Table 1: Temperature measurements with different thermometers. All temperature values in °C.

Set-point	Greisinger	Voltcraft	Fiber 1	Fiber 2	Fiber 3	Fiber 4
0	0.1 ± 0.2	0.2 ± 0.5	-0.1 ± 0.1	0.0 ± 0.1	0.0 ± 0.2	-0.1 ± 0.1
15	15.1 ± 0.2	15.0 ± 0.5	15.6 ± 0.1	15.6 ± 0.2	15.7 ± 0.2	15.7 ± 0.2
16	16.1 ± 0.2	16.0 ± 0.5	16.7 ± 0.2	16.8 ± 0.1	16.8 ± 0.2	16.9 ± 0.2
17	17.1 ± 0.2	16.9 ± 0.5	17.8 ± 0.1	17.8 ± 0.1	17.9 ± 0.2	17.9 ± 0.2
18	18.1 ± 0.2	17.8 ± 0.5	18.8 ± 0.1	18.9 ± 0.2	18.9 ± 0.2	19.0 ± 0.2
19	19.1 ± 0.2	18.8 ± 0.5	19.9 ± 0.2	19.9 ± 0.1	19.9 ± 0.2	20.0 ± 0.1
20	20.1 ± 0.2	19.8 ± 0.5	20.9 ± 0.1	20.9 ± 0.2	21.0 ± 0.1	20.9 ± 0.1
21	21.1 ± 0.2	20.8 ± 0.5	21.9 ± 0.2	22.0 ± 0.2	22.0 ± 0.1	22.0 ± 0.2
22	22.1 ± 0.2	21.8 ± 0.5	22.9 ± 0.2	22.9 ± 0.2	23.0 ± 0.1	23.0 ± 0.1
23	23.1 ± 0.2	22.8 ± 0.5	23.9 ± 0.2	23.9 ± 0.1	23.9 ± 0.1	24.1 ± 0.2
24	24.1 ± 0.2	23.8 ± 0.5	24.9 ± 0.1	24.9 ± 0.1	25.0 ± 0.1	25.0 ± 0.1
25	25.1 ± 0.2	24.8 ± 0.5	25.8 ± 0.1	25.9 ± 0.2	25.9 ± 0.1	26.0 ± 0.2
26	26.1 ± 0.2	25.8 ± 0.5	26.9 ± 0.1	26.9 ± 0.2	27.0 ± 0.1	27.0 ± 0.1
27	27.1 ± 0.2	26.8 ± 0.5	27.8 ± 0.1	27.9 ± 0.2	27.9 ± 0.1	27.9 ± 0.1
28	28.1 ± 0.2	27.8 ± 0.5	28.8 ± 0.1	28.9 ± 0.1	29.0 ± 0.1	28.9 ± 0.1
29	29.1 ± 0.2	28.8 ± 0.5	29.8 ± 0.2	29.9 ± 0.2	29.9 ± 0.1	30.0 ± 0.2
30	30.1 ± 0.2	29.8 ± 0.5	30.7 ± 0.1	30.9 ± 0.2	31.0 ± 0.2	30.9 ± 0.1
31	31.1 ± 0.2	30.8 ± 0.5	31.8 ± 0.1	31.8 ± 0.1	31.9 ± 0.1	31.9 ± 0.1
32	32.1 ± 0.2	31.8 ± 0.5	32.8 ± 0.1	32.8 ± 0.2	32.9 ± 0.1	32.9 ± 0.1
33	33.1 ± 0.2	32.8 ± 0.5	33.8 ± 0.2	33.9 ± 0.1	33.9 ± 0.1	34.0 ± 0.2
34	34.1 ± 0.2	33.8 ± 0.5	34.8 ± 0.1	34.8 ± 0.2	35.0 ± 0.2	35.0 ± 0.2
35	35.1 ± 0.2	34.8 ± 0.5	35.8 ± 0.1	35.8 ± 0.1	35.9 ± 0.1	35.9 ± 0.1

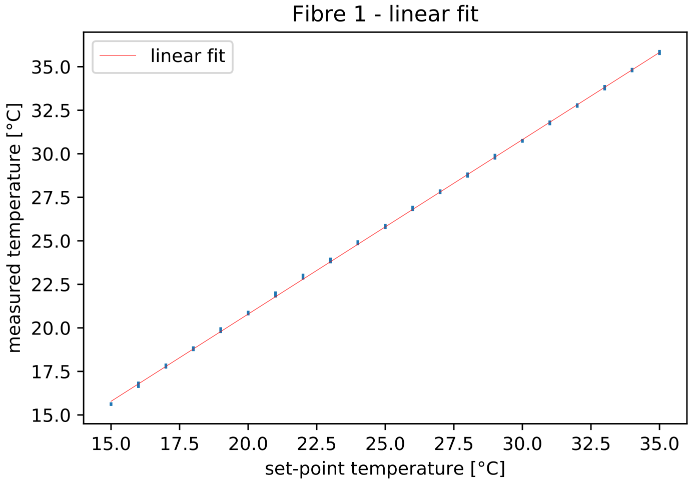

Figure 4: A linear function is fitted to the data of fiber 1.

Table 2: Offset of the fibers to the set-point values

Set-point [°C]	Fiber 1 [°C]	Fiber 2 [°C]	Fiber 3 [°C]	Fiber 4 [°C]
0	-0.1 ± 0.1	0.0 ± 0.1	0.0 ± 0.2	-0.1 ± 0.1
15	0.6 ± 0.1	0.6 ± 0.2	0.7 ± 0.2	0.7 ± 0.2
16	0.7 ± 0.2	0.8 ± 0.1	0.8 ± 0.2	0.9 ± 0.2
17	0.8 ± 0.1	0.8 ± 0.1	0.9 ± 0.2	0.9 ± 0.2
18	0.8 ± 0.1	0.9 ± 0.2	0.9 ± 0.2	1.0 ± 0.2
19	0.9 ± 0.2	0.9 ± 0.1	0.9 ± 0.2	1.0 ± 0.1
20	0.9 ± 0.1	0.9 ± 0.2	1.0 ± 0.1	0.9 ± 0.1
21	0.9 ± 0.2	1.0 ± 0.2	1.0 ± 0.1	1.0 ± 0.2
22	0.9 ± 0.2	0.9 ± 0.2	1.0 ± 0.1	1.0 ± 0.1
23	0.9 ± 0.2	0.9 ± 0.1	0.9 ± 0.1	1.1 ± 0.2
24	0.9 ± 0.1	0.9 ± 0.1	1.0 ± 0.1	1.0 ± 0.1
25	0.8 ± 0.1	0.9 ± 0.2	0.9 ± 0.1	1.0 ± 0.2
26	0.9 ± 0.1	0.9 ± 0.2	1.0 ± 0.1	1.0 ± 0.1
27	0.8 ± 0.1	0.9 ± 0.2	0.9 ± 0.1	0.9 ± 0.1
28	0.8 ± 0.1	0.9 ± 0.1	1.0 ± 0.1	0.9 ± 0.1
29	0.8 ± 0.2	0.9 ± 0.2	0.9 ± 0.1	1.0 ± 0.2
30	0.7 ± 0.1	0.9 ± 0.2	1.0 ± 0.2	0.9 ± 0.1
31	0.8 ± 0.1	0.8 ± 0.1	0.9 ± 0.1	0.9 ± 0.1
32	0.8 ± 0.1	0.8 ± 0.2	0.9 ± 0.1	0.9 ± 0.1
33	0.8 ± 0.2	0.9 ± 0.1	0.9 ± 0.1	1.0 ± 0.2
34	0.8 ± 0.1	0.8 ± 0.2	1.0 ± 0.2	1.0 ± 0.2
35	0.8 ± 0.1	0.8 ± 0.1	0.9 ± 0.1	0.9 ± 0.1

Table 3: Fitting parameters of linear fits to fiber temperature data

slope	y-intercept [°C]
1.002 ± 0.003	0.75 ± 0.07
1.00 ± 0.03	0.88 ± 0.07
1.00 ± 0.02	0.93 ± 0.06
1.001 ± 0.003	0.92 ± 0.07

Table 4: Measured temperatures at the beginning of the experiment (low field) and at the end (high field).

beginning [°C]	end [°C]
0.0 ± 0.1	0.0 ± 0.1
0.0 ± 0.1	0.0 ± 0.1
0.0 ± 0.2	0.0 ± 0.1
0.1 ± 0.1	0.0 ± 0.1

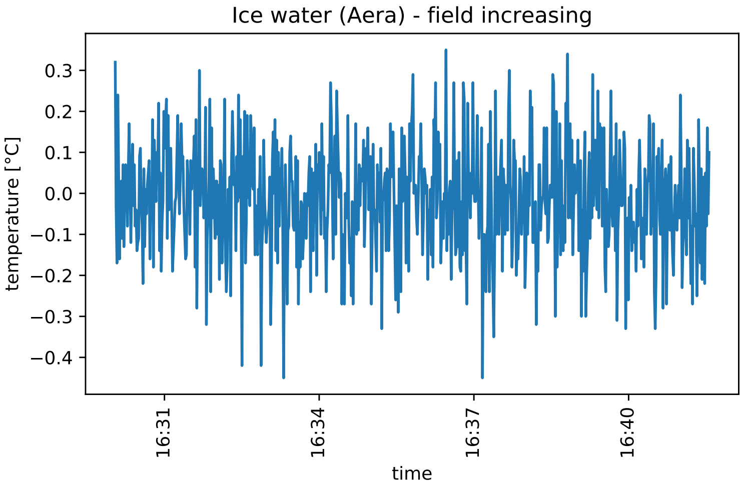

Figure 5: The temperature (recorded with fiber 1) is plotted against time. During the experiment, the insulated container containing the ice water with the fibers was moved into the MR scanner.

5 Discussion and Outlook

In the first part of the experiment, the accuracy of the Luxtron temperature device was examined. The measurement series confirmed that the device showed an offset to the set-point temperature value, which can be interpreted as a systematic error. This problem is not easy to solve by a two-point calibration. The origin of the offset needs to be further investigated.

The second part of the experiment aimed to investigate a potential influence of magnetic fields on the Luxtron device. No evidence could be collected for such a behavior.
