# Supplementary material for: Temperature and concentration calibration of aqueous polyvinylpyrrolidone (PVP) solutions for isotropic diffusion MRI phantoms
Source: PLoS One. 2017 Jun 19;12(6):e0179276. doi: 10.1371/journal.pone.0179276 (PMC5476261; doi:10.1371/journal.pone.0179276)
Supplement: S2 File — (DOCX) [file pone.0179276.s002.docx]

Supplemental Material 2: Separate Evaluation for 1.5 T and 3 T

Tables A1 and A2 provide the fitted parameters $c_{1}$ and $c_{2}$ using only the data acquired at 1.5 T. Tables A3 and A4 provide the fitted parameters $c_{1}$ and $c_{2}$ using only the data acquired at 3 T.

Tab. A1: Measurements for K30 at 1.5 T: Fit parameters describing the dependency of $D_{\mathrm{app}}$ on the temperature (see Eq. (2)) for K30 and data acquired at 1.5 T. 95% confidence intervals are stated in brackets.

| $c_{\mathrm{PVP}}$ [% (w/w)] | $c_{1}$ [µm²/ms] | $c_{2}$ [1/K] |
| --- | --- | --- |
| 0 | 2.063 (2.057, 2.069) | 0.02654 (0.02604, 0.02703) |
| 10 | 1.597 (1.596, 1.598) | 0.0253 (0.02514, 0.02545) |
| 20 | 1.200 (1.198, 1.202) | 0.02717 (0.02686, 0.02748) |
| 30 | 0.8357 (0.8337, 0.8376) | 0.02904 (0.02862, 0.02946) |
| 40 | 0.5419 (0.5398, 0.5441) | 0.03189 (0.03118, 0.03259) |
| 50 | 0.3262 (0.3243, 0.3282) | 0.03245 (0.03138, 0.03351) |

Tab. A2: Measurements for K90 at 1.5 T: Fit parameters describing the dependency of $D_{\mathrm{app}}$ on the temperature (see Eq. (2)) for K90 and data acquired at 1.5 T. 95% confidence intervals are stated in brackets.

| $c_{\mathrm{PVP}}$ [% (w/w)] | $c_{1}$ [µm²/ms] | $c_{2}$ [1/K] |
| --- | --- | --- |
| 10 | 1.610 (1.608, 1.613) | 0.02391 (0.02361, 0.02421) |
| 20 | 1.185 (1.182, 1.187) | 0.02474 (0.02434, 0.02513) |
| 30 | 0.8088 (0.8068, 0.8109) | 0.02825 (0.02779, 0.02871) |

Tab. A3: Measurements at 3 T: Fit parameters describing the dependency of $D_{\mathrm{app}}$ on the temperature (see Eq. (2)) for K30 and data acquired at 3 T. 95% confidence intervals are stated in brackets.

| $c_{\mathrm{PVP}}$ [% (w/w)] | $c_{1}$ [µm²/ms] | $c_{2}$ [1/K] |
| --- | --- | --- |
| 0 | 2.038 (2.034, 2.041) | 0.02583 (0.02555, 0.0261) |
| 10 | 1.589 (1.587, 1.590) | 0.02533 (0.02519, 0.02548) |
| 20 | 1.191 (1.189, 1.192) | 0.02788 (0.02771, 0.02804) |
| 30 | 0.8453 (0.8437, 0.847) | 0.02995 (0.02967, 0.03023) |
| 40 | 0.5557 (0.5541, 0.5572) | 0.03287 (0.03248, 0.03326) |
| 50 | 0.3450 (0.3413, 0.3486) | 0.03318 (0.03167, 0.03469) |

Tab. A4: Measurements for K90 at 3 T: Fit parameters describing the dependency of $D_{\mathrm{app}}$ on the temperature (see Eq. (2)) for K90 and data acquired at 3 T. 95% confidence intervals are stated in brackets.

| $c_{\mathrm{PVP}}$ [% (w/w)] | $c_{1}$ [µm²/ms] | $c_{2}$ [1/K] |
| --- | --- | --- |
| 10 | 1.579 (1.577, 1.581) | 0.02528 (0.02511, 0.02546) |
| 20 | 1.181 (1.179, 1.183) | 0.02606 (0.02578, 0.02634) |
| 30 | 0.8166 (0.8153, 0.8178) | 0.02958 (0.02935, 0.02981) |
