# Supplementary material for: Temperature and concentration calibration of aqueous polyvinylpyrrolidone (PVP) solutions for isotropic diffusion MRI phantoms
Source: PLoS One. 2017 Jun 19;12(6):e0179276. doi: 10.1371/journal.pone.0179276 (PMC5476261; doi:10.1371/journal.pone.0179276)
Supplement: S4 File — (DOCX) [file pone.0179276.s004.docx]

**Supplemental Material 4 - Assessment of image blurring and Gibbs ringing**

To suppress the effect of „long-range“ sinc-like Gibbs ringing, the vendor-provided image filter had been set to “strong”. This filter process may results in an increased image blurring, which had not been assessed in detail with resolution phantoms. The blurring can, however, be roughly estimated from the images themselves.

Figure S1 shows one high and one low resolution image acquired with $b$=0 at 1.5 T. The upper row shows the complete image, the second one shows an image section containing one of the bins. It is visually perceivable that a Gibbs ringing induced signal overshoot of about one voxel size is still present. The lower row in Fig. S1 shows the signal profile along the red line plotted in the images. At the boundary of the phantom, the signal rises from noise level to full level within two voxels. Moreover, the boundaries of the bins can be clearly appreciated.

Given that the ROIs were defined with a safety margin from the bin boundary (see Fig. 2), we estimate the effect of Gibbs ringing and filter-induce blurring to be acceptably small.


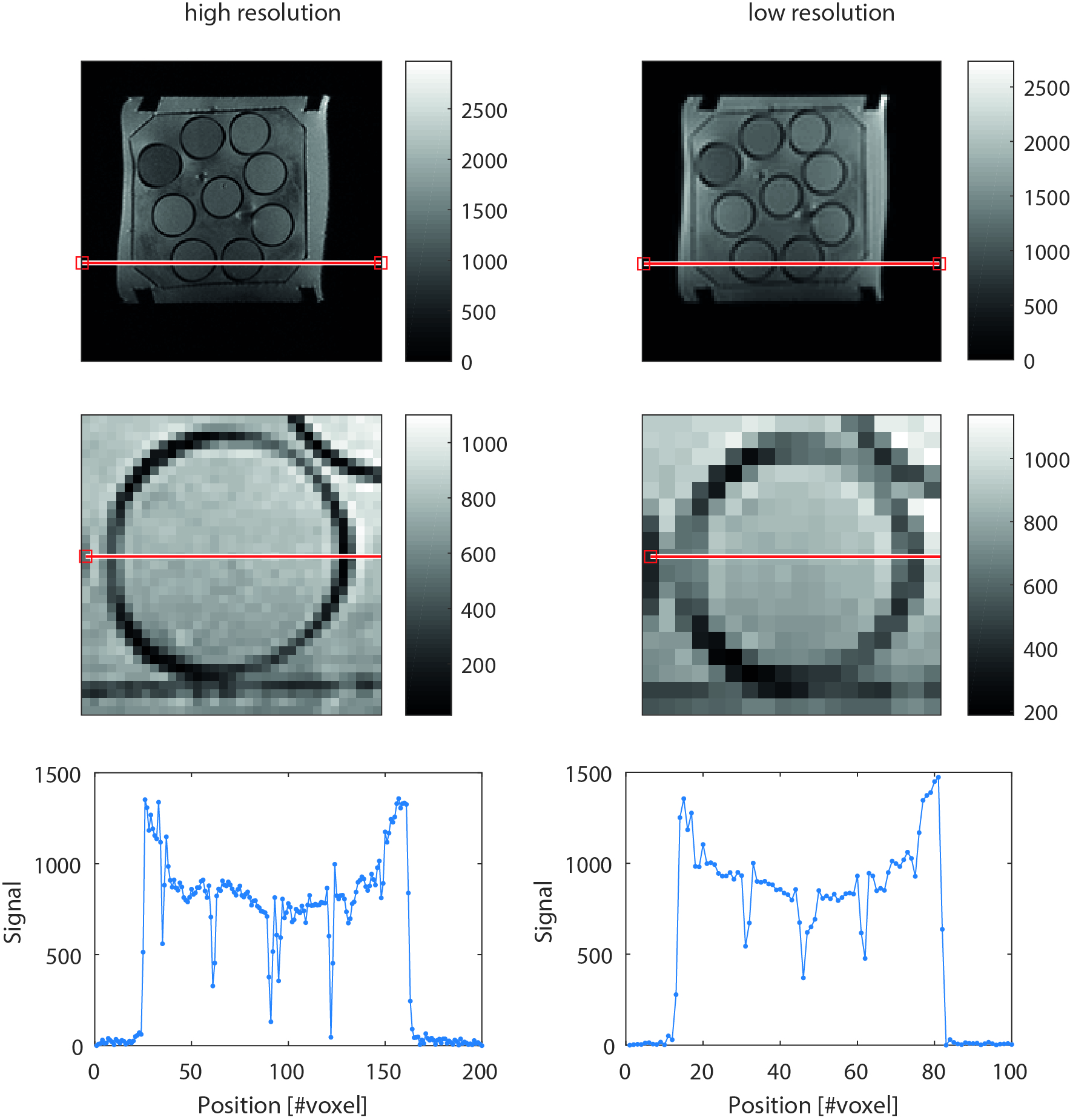


Figure S1. Assessment of the Gibbs ringing. The upper row displays $b$=0 images acquired at 1.5 T. The middle row displays image sections containing one of the bins. Images are min-max normalized. The lower row shows the signal profile along the red line displayed in the images.
